# Heart failure with preserved ejection fraction: The role of intravascular volumes and body composition in exercise-induced progenitor cell mobilization^1^

**DOI:** 10.1016/j.ijcha.2026.101967

**Published:** 2026-06-29

**Authors:** Julia M. Kröpfl, Raphael Schoch, Benedikt Gasser, Luisa Prechtl, Hans-Jürgen Gruber, Rupprecht Wick, Thomas Dieterle, Marijke Brink, Arno Schmidt-Trucksäss

**Affiliations:** a Division of Sport and Exercise Medicine, Department of Sport, Exercise and Health, University of Basel, Grosse Allee 6, 4052 Basel, Switzerland; b Clinical Institute of Medical and Chemical Laboratory Diagnostics, Medical University of Graz, Auenbruggerplatz 15, 8036 Graz, Austria; c Clinic Arlesheim, Division of Cardiology, Arlesheim, Switzerland; d Department of Clinical Research, University of Basel, 4031 Basel, Switzerland; e Merian Iselin Clinic, Basel, Switzerland; f Cardiobiology, Department of Biomedicine, University of Basel, Hebelstrasse 20, 4031 Basel, Switzerland

**Keywords:** Circulating progenitor cell, Intravascular volumes, Chronic exercise, HFpEF

## Abstract

**Background and Aims:** Whether exercise training induces plasma volume (PV) expansion and distorts circulating progenitor cell (CPC) assessment in heart failure with preserved ejection fraction (HFpEF) remains unclear, particularly in women, who might have lower CPC concentrations and higher cardiovascular risk due to female-specific risk factors. This study investigated sex-specific differences, skeletal muscle mass (SMM)-normalized baseline values, and exercise-induced changes in intravascular volumes, CPCs, and erythropoietin (EPO). **Methods:** HFpEF patients completed a 3-months strength–endurance training. Blood volume (BV), PV, red blood cell volume (RBCV), and hemoglobin mass (Hb-mass) were assessed using carbon-monoxide rebreathing, CPCs by flow cytometry, EPO by ELISA. CPC concentrations were adjusted for BV. Training effects, sex interactions, and associations between SMM-normalized CPCs and EPO were examined. **Results:** At baseline, women had comparable BV, PV, RBCV, Hb-mass, and EPO to men when normalized to SMM (all *p* > 0.05, uncorrected). After BV adjustment, women showed lower total CPC counts (uncorrected *p* = 0.031), but this difference disappeared after SMM normalization (uncorrected *p* = 0.160). Exercise training showed no evidence of affecting SMM-normalized intravascular volumes, Hb-mass, EPO, or BV-(un)adjusted CPC concentrations, with no interaction effects of sex (all *p* > 0.05, uncorrected). Before SMM normalization, exploratory analysis revealed PV changes being inversely associated with CPC concentration (ρ = −0.57, *p* = 0.044), but not thereafter. CPC relationships to EPO were non-significant (both *p* > 0.05). **Conclusions:** Sex differences in CPCs were evident after adjusting for BV but disappeared after SMM normalization. Exercise training did not increase CPCs, but individual responses depended on CPC expression. Findings require validation in larger HFpEF cohorts.

## Graphical abstract

**Figure:**
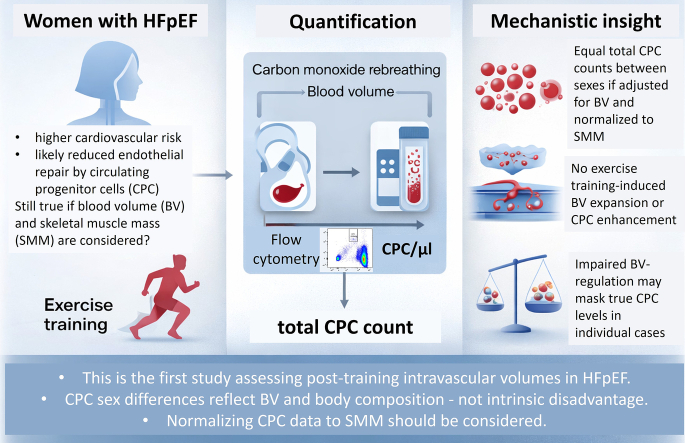

## 1 Introduction

In decompensated heart failure with preserved ejection fraction (HFpEF), neurohormonal activation and systemic congestion play central roles [1]. Enhanced sodium and water retention increase plasma volume (PV) and red blood cell volume (RBCV), leading to expansion of total blood volume (BV) [2] and the possible development of dilutional anemia, characterized by reduced hemoglobin (Hb) and hematocrit. In contrast, stable HFpEF patients receiving standard pharmacotherapy often exhibit hypovolemia, with PV comparable to healthy controls but reduced RBCV and total BV, indicating true (non-dilutional) anemia due to a decreased Hb-mass [3]. Moreover, endocrine systems regulating fluid homeostasis and hematopoiesis are overactivated [3].

Exercise training likely modulates this process. In healthy individuals, regular aerobic exercise induces a physiological PV expansion through repeated exercise-induced fluid shifts and increased plasma protein content, which promote fluid retention. This expansion contributes to increased ventricular filling (preload) and thereby enhances stroke volume via the Frank–Starling mechanism, ultimately improving oxygen delivery [4]. This adaptive hemodilution reflects improved cardiovascular efficiency. Whether a similar PV expansion occurs in HFpEF remains uncertain, especially if values are normalized to skeletal muscle mass (SMM) [5], yet clarifying this response is essential for accurate interpretation of laboratory results.

Circulating progenitor cells (CPCs) serve as a complementary marker of vascular repair in HFpEF, where endothelial dysfunction drives disease progression [6]. Reduced CPCs indicate impaired endothelial integrity and are linked to worse outcomes [7]. Women, who are at higher cardiovascular risk across the lifespan due to female-specific risk factors [8], often exhibit lower CPC concentrations, indicating reduced endothelial repair capacity [9]. Whether this sex-related difference also persists in HFpEF remains unknown. Exercise training enhances CPC mobilization in healthy individuals [10], whereas in HFpEF it did not show the same effect [11], yet hypovolemia from standard pharmacotherapy or exercise-induced hemodilution could distort measured cell concentrations. In addition, SMM – via the release of chemoattractants - may further influence CPC levels by mobilizing cells from bone-marrow or vascular niches [12]. Misinterpreting these volume-related changes - particularly when considering sex-specific body composition differences - may lead to inaccurate assessment of disease severity or therapeutic response when evaluating CPC data in HFpEF. Therefore, this study aims to determine whether exercise training in HFpEF modifies the magnitude of sex-specific intravascular volume responses.

## 2 Methods

We investigated individual baseline and exercise training-induced CPC responses in patients with HFpEF normalized to SMM, expressed both as cell concentration and as total circulating cell count adjusted for BV. We further examined their association with changes in erythropoietin (EPO) regulating hematopoiesis. Baseline hypovolemia may artifactually elevate measured CPC concentrations, whereas training-induced hemodilution may obscure true improvements in regenerative potential. Therefore, total SMM-normalized CPC count, rather than concentration, is expected to more accurately reflect endocrine changes by correlating with EPO.

As part of a larger clinical study (NCT03184311; EKNZ-number 2019–00188), patients with HFpEF completed 3-month strength-endurance training [13]. Medical records at the Department of Sport, Exercise and Health (DSBG, University of Basel, Switzerland) and the Division of Cardiology (Clinic Arlesheim, Switzerland) were reviewed to identify patients treated for HFpEF according to the 2019 diagnostic criteria [14]. In brief, inclusion required LVEF >50%, elevated NT-proBNP, and echocardiographic abnormalities (e.g., LAVI >34 mL/m^2^, LV hypertrophy [LVMI >95 g/m^2^ in women or > 115 g/m^2^ in men], or E/E′ >13 with mean E′ <9 cm/s). All participants were on stable medication for at least four weeks and showed no signs of acute cardiac decompensation. Due to severe recruitment challenges during the COVID-19 pandemic, patients with HFpEF and NYHA class I were also included, deviating from the original protocol [13]. This adjustment was justified because NYHA class is not a strict diagnostic criterion for HFpEF; current guidelines instead emphasize objective echocardiographic, hemodynamic, and biomarker-based measures rather than symptom severity alone [14], [15], [16], [17], [18].

In a subset of recruited patients (*n* = 33; 12 males), 15 mL of venous blood was collected from the cubital vein at baseline (PRE, n = 33) and, after exercise training (POST, *n* = 29). Please find the baseline characteristics of the study population in Table 1. Patients were selected based on the availability of blood data; however, the final sample was quite balanced across exercise training modalities: HIT *n* = 15 (4 males); MCT *n* = 14 (6 males). As no differences between training modalities in CPC data had been observed previously [11], further analyses were conducted without distinguishing between modalities. Please find a comparison of baseline variables between training modalities in Supplementary Table 1.

**Table 1:** Baseline characteristics of study population separated by sex.

Characteristic		Total (n=33)			males (n=12)		females (n=21)	
Age (at inclusion) *(mean (SD) [n])*		72.0	(9.0) [33]		68.3	(12.4) [12]	73.8	(6.6) [21]
Body mass index *(mean (SD) [n])*		26.0	(5.4) [33]		27.0	(2.6) [12]	25.4	(6.5) [21]
Systolic BP (mmHg) *(mean (SD) [n])*		137	(22) [33]		134	(16) [12]	139	(25) [21]
Diastolic BP (mmHg) *(mean (SD) [n])*		85	(11) [33]		86	(9) [12]	85	(12) [21]
NT-proBNP *(median (IQR) [n])*		235	(140, 401) [32]		137	(65, 466) [11]	240	(187, 388) [21]
NYHA-class *(No. (%))*	1	8	(24)		2	(17)	6	(29)
	2	15	(46)		8	(67)	7	(33)
	3	10	(30)		2	(17)	8	(38)
								
*Cardiovascular risk factors (No. (%))*								
Hypertension		24	(73)		10	(83)	14	(67)
Hyperlipidemia		13	(39)		6	(50)	7	(33)
Diabetes		2	(6)		1	(8)	1	(5)
Never smoked		16	(49)		4	(33)	12	(57)
Ex-Smoker		14	(42)		6	(50)	8	(38)
Current Smoker		3	(9)		2	(17)	1	(5)
								
*Medication (No. (%))*								
ACE/ARB		21	(64)		10	(83)	11	(52)
Other antihypertensive medication		10	(30)		7	(58)	3	(14)
β-Blockers		4	(12)		3	(25)	1	(5)
Diuretics		11	(33)		6	(50)	5	(24)
Cholesterol-lowering drugs		8	(24)		4	(33)	4	(19)
Antiplatelet		6	(18)		3	(25)	3	(14)
								
*Comorbidities (No. (%))*								
Coronary artery disease		7	(21)		5	(42)	2	(10)
Atrial fibrillation		9	(27)		4	(33)	5	(24)
COPD		4	(12)		1	(8)	3	(14)
Chronic kidney disease		1	(3)		1	(8)	0	(0)
Stroke		1	(3)		0	(0)	1	(5)

Data are presented as mean (standard deviation) or median (IQR)*. BP* blood pressure, *NT-proBNP* B-type natriuretic peptide, *NYHA* New York Heart Association classification, *ACE* angiotensin-converting enzyme inhibitor, *ARB* angiotensin receptor blocker, *COPD* chronic obstructive pulmonary disease.

Circulating progenitor cell concentrations (CD34+CD45dim) were quantified by flow cytometry as previously described [19]. Briefly, after mononuclear cell isolation, cells were stained with CD34, CD45, CD31 antibodies and a live/dead (Aqua) as well as an apoptosis marker (Annexin-V). Cell analysis was done on a Cytoflex device (Beckman Coulter, Nyon, Switzerland). After gating for mononuclear cells and doublets exclusion, cells were analyzed for CD34+ and CD45dim expressions with a low side scatter. Total BV, RBCV, and PV were determined using the carbon-monoxide rebreathing technique with the Detalo Performance™ device (Detalo Health ApS, Hørsholm, Denmark) [3], [20]. After 10 min of supine rest, baseline %HbCO and Hb concentration were assessed from fingertip capillary blood (RapidPoint 500e, Siemens Healthineers, Erlangen, Germany). Patients then inhaled oxygen and rebreathed a bodyweight-adjusted CO bolus for 6 min. After equilibration, %HbCO and Hb were remeasured, and Hb-mass was calculated from the rise in %HbCO and the remaining CO in the system. EPO was assessed by ELISA (Invitrogen, Cat#BMS2035–2). Total CPC counts were derived by multiplying the respective cell concentrations by the corresponding BV. All parameters were normalized to each patient's individual SMM, which was measured by bioimpedance (InBody 720, InBody Co. Ltd., Seoul, South Korea). No patient showed signs of clinically relevant edema, as all extracellular water-to-total body water ratios were ≤ 0.41, suggesting absence of significant fluid overload. Data are presented by sex, shown as means (standard deviations), median (IQR) or as individual values.

A literature-based [9] a priori sample size calculation indicated that 11 males and 22 females were required to detect a significant sex difference in CPCs (two-tailed independent *t*-test; effect size = 1.098; α = 0.05; power = 0.80; allocation ratio = 2). Furthermore, 24 participants were needed to detect a potential training-induced effect on CPCs in healthy individuals (two-tailed paired t-test; effect size = 0.611; α = 0.05; power = 0.80) [21]. Between-group differences at baseline and post-intervention were analyzed using independent *t*-tests or Mann-WhitneyU tests, as appropriate. Two-way repeated-measures ANOVA (factors: time, sex) was applied to assess training-induced changes. No correction for multiple comparisons was performed, given the exploratory nature of the analyses. Associations between parameters were evaluated using Pearson or Spearman correlation coefficients, depending on data distribution and normality assumptions. Influential observations were assessed using Cook's distance. Statistical significance was defined as two-sided *p* < 0.05. Effect sizes (η_p_^2^) are additionally indicated if F-statistics are approaching conventional significance thresholds.

## 3 Results

**Fig. 1:**
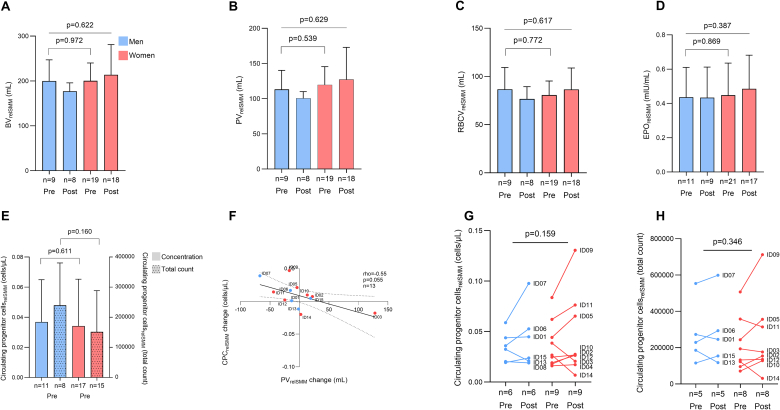
Intravascular volumes, erythropoietin, and circulating progenitor cell responses to exercise training in HFpEF. Baseline blood volume (BV, A), plasma volume (PV, B), red blood cell volume (RBCV, C), and erythropoietin (EPO, D) showed no evidence of a meaningful difference between sexes when normalized to SMM (*p* = 0.539–0.972). Exercise training did not alter any of these parameters (*p* = 0.387–0.629) with no sex-specific interaction effects (*p* = 0.067–0.979). Sex differences in baseline circulating progenitor cells (CPCs, E) became apparent only after accounting for BV (*p* = 0.031, data not shown) but vanished after normalizing total CPC count to SMM (*p* = 0.160). Before SMM normalization, exploratory analysis suggested an inverse association between PV changes and CPC concentration (ρ = −0.57, *p* = 0.044; data not shown), which attenuated after normalization (ρ = −0.55, *p* = 0.055). Given the small sample size, this finding should be considered hypothesis-generating. Exercise training did not notably increase CPC concentrations (*p* = 0.159, G), nor total circulating CPC counts (BV-adjusted and normalized to SMM, *p* = 0.346, H), with no interaction effect of sex (*p* = 0.937 and *p* = 0.607, respectively).

Baseline BV (Fig. 1A), PV (Fig. 1B), RBCV (Fig. 1C), EPO (Fig. 1D**)**, and Hb-mass normalized to SMM demonstrated no meaningful difference between females and males (t(26) = −0.036, *p* = 0.972, *n* = 28; t(26) = −0.622, *p* = 0.539, n = 28; U = 79, *p* = 0.772, n = 28; t(30) = −0.166, *p* = 0.869, *n* = 32; and t(26) = 0.837, *p* = 0.410, n = 28; respectively). When comparing baseline CPC concentrations and total circulating CPC counts between sexes, the lower regenerative potential in women was only evident after solely accounting for volume status (t(21) = 2.309, *p* = 0.031, *n* = 23), but vanished after normalizing total CPC count to SMM (t(21) = 1.456, *p* = 0.160, n = 23, Fig. 1E). CPC concentrations normalized to SMM also displayed no notable divergence between sexes (U = 82, *p* = 0.611, *n* = 28; Fig. 1E).

Exercise training showed no evidence of altering Hb-mass (F(1, 24) = 0.002, *p* = 0.967), BV, PV, RBCV, or EPO concentrations normalized to SMM (F(1, 24) = 0.250, *p* = 0.622; F(1, 24) = 0.240, *p* = 0.629; F(1, 24) = 0.257, *p* = 0.617; and F(1, 24) = 0.776, *p* = 0.387; respectively; all *n* = 26, Fig. 1A-D). No significant sex-specific interaction effects were observed for the aforementioned variables (F(1, 24) = 2.904, *p* = 0.101, η_p_^2^  = 0.088; F(1, 24) = 2.327, *p* = 0.140; F(1, 24) = 1.642, *p* = 0.212; F(1, 24) = 3.689, *p* = 0.067, η_p_^2^ = 0.133; and F(1, 24) = 0.001, *p* = 0.979, respectively), although effect sizes for Hb-mass and RBCV were medium to large.

Correlations between absolute post-training changes in PV and changes in CPC concentrations appeared inversely associated before normalization to SMM, suggesting a potential relationship (ρ = −0.57, *p* = 0.044; *n* = 13, data not shown), but not thereafter (ρ = −0.55, *p* = 0.055, n = 13, Fig. 1F). No observations exceeded the predefined Cook's distance threshold of 4/n; however, the results should be considered exploratory. At the group level, exercise training showed no evidence of an increase in CPC concentrations (F(1, 13) = 2.233, *p* = 0.159; Fig. 1G, *n* = 15) or total circulating CPC counts adjusted for BV and normalized to SMM (F(1, 11) = 0.968, *p* = 0.346, n = 13; Fig. 1H), with no interaction effect of sex (F(1, 13) = 0.006, *p* = 0.937; and F(1, 11) = 0.281, *p* = 0.607; respectively). Furthermore, training-induced changes in SMM-normalized CPC concentrations and total CPC counts were not significantly correlated with relative changes in SMM-normalized EPO (*r* = 0.33, *p* = 0.246, *n* = 14; rho = 0.50, *p* = 0.095, *n* = 12; respectively). However, due to the small sample size, the correlation results are hypothesis-generating rather than confirmatory.

## 4 Discussion

This is the first study to describe an absence of change in intravascular volume normalized to SMM and its potential impact on CPC assessment following exercise training in HFpEF.

Notably, individual responses to exercise training varied depending on how CPCs were expressed. When total circulating CPC counts were considered instead of concentrations, inter-individual differences became apparent that were otherwise obscured when volume status was disregarded, despite taking body composition differences into account. For example, in patients ID11 and ID03, CPC concentrations indicated an apparent increase and decrease in regenerative capacity, respectively, whereas total CPC counts either declined or remained stable following training (Fig. 1G, H). These observed directional discrepancies may be clinically meaningful but could also reflect measurement noise in the assessment methods. A resulting hypothesis is that interpretation of CPC data in HFpEF may require consideration of both cell concentration and total circulating cell counts, particularly when comparing women and men or evaluating responses to exercise training. If intravascular volumes differ by sex and are dynamically modulated by training status, concentration-based measures (e.g., cells·mL^−1^) can be confounded by hemodilution or hemoconcentration effects. Notably though, women with HFpEF show no evidence of a lower PV at baseline when normalized to SMM, which indicates a comparable regenerative capacity between sexes expressed as cell concentration or total cell count. For the first time we observed no indication of SMM-normalized PV expansion in either sex following exercise training, resulting in a non-dilutional CPC concentration reflecting true circulating progenitor cell availability. In contrast to the well-described intravascular volume expansion observed with exercise training in healthy individuals [4] - with women sometimes exhibiting greater relative increases - our findings in HFpEF may reflect impairment of the cardiac, vascular, renal, and neurohormonal pathways that normally regulate volume expansion and hematopoiesis [1], [3], likely to a similar extent in both sexes. Although untested, some potential mechanisms may be hypothesized: Altered renal receptor signaling or ineffective erythropoiesis may contribute to blunted adaptive responses and limit further physiological expansion. [3], [22]. Concomitant pharmacological therapies commonly used in HFpEF, particularly diuretics and neurohormonal modulators, may attenuate or counteract exercise-induced PV and BV expansion [23] by sodium excretion and/or water loss limiting preload reserve. In this context, exercise training may be insufficient to normalize dysregulated EPO signaling or renal sodium and fluid handling. Or EPO responsiveness may be blunted despite preserved or elevated levels, potentially due to bone marrow insensitivity, or chronic low-grade inflammation as shown in HFrEF [24]. Alternatively, exercise training may predominantly alter venous capacitance and BV distribution, shifting blood toward the stressed compartment without increasing total PV or BV, as previously suggested following acute exercise [25]. However, none of these mechanisms were assessed in the present study; thus, the mechanistic basis underlying the null findings remains unclear. Furthermore, nominally significant findings should be interpreted cautiously because no correction for multiple comparisons was applied.

Some limitations should be acknowledged. Due to the absence of a non-exercising control group the null findings for exercise training cannot be distinguished from e.g. natural temporal variation, regression to the mean, or pharmacotherapy-related fluctuations. Also, mechanistic mediators such as renal sodium handling, plasma protein synthesis or bone marrow responsiveness were not directly assessed. Moreover, differences in disease severity (NYHA class I versus II/III) may have biased the results toward null findings. However, no NYHA class I patients were included in the analysis of training-induced effects on CPCs. Missing data resulted from several factors: dropouts (n_post_ = 4), patients declining the (repeated) rebreathing measurement (n_pre_ = 2, nₚₒₛₜ = 1) or failed rebreathing measurements (n_pre_ = 3, nₚₒₛₜ = 1), difficulties with blood withdrawal (nₚᵣₑ = 3, n_post_ = 1), issues during MNC isolation (nₚᵣₑ = 1), exclusion of extreme outliers in flow cytometry analysis (nₚᵣₑ = 1), EPO values below the detection limit (nₚₒₛₜ = 1), and lack of available serum samples (nₚₒₛₜ = 1). Missing data resulted in reduced statistical power for some analyses and may have introduced selection bias; thus, findings should be interpreted as exploratory and correlation results as hypothesis-generating.

## 5 Conclusion

The absence of evidence for exercise training-induced PV or BV expansion in HFpEF suggests that impaired volume regulation may mask CPC mobilization in individual cases, emphasizing the possible importance of accounting for hemodynamic factors such as BV when evaluating CPC responses. The findings further suggest that sex differences in CPCs may largely reflect differences in BV and body composition rather than intrinsic disadvantage. Therefore, normalizing CPC data to SMM should be considered for the assessment of sex differences and training-induced adaptations, improving data precision and interpretability in future studies.

## Author’s contribution

The experiments were performed at the Department of Sport, Exercise and Health (DSBG) and the Department of Biomedicine, University of Basel, Switzerland. JMK, MB and AST were responsible for the conception or design of the work. JMK, RS, BAG, LP, HJG, and RW were responsible for data acquisition and analysis. JMK, RS, BAG, LP, HJG, RW, TD, MB, and AST drafted the work or revised it critically for important intellectual content. All authors were responsible for interpretation of data for the work. In addition, all authors approved the final version of the manuscript, agreed to be accountable for all aspects of the work in ensuring that questions related to the accuracy or integrity of any part of the work were appropriately investigated and resolved. All persons designated as authors qualify for authorship, and all those who qualify for authorship are listed.

## CRediT authorship contribution statement

**Julia M. Kröpfl:** Writing – original draft, Visualization, Methodology, Investigation, Funding acquisition, Conceptualization. **Raphael Schoch:** Writing – review & editing, Supervision, Project administration, Methodology, Investigation. **Benedikt Gasser:** Writing – review & editing, Supervision, Project administration. **Luisa Prechtl:** Writing – review & editing, Methodology, Investigation. **Hans-Jürgen Gruber:** Writing – review & editing, Validation, Methodology, Investigation, Formal analysis. **Rupprecht Wick:** Writing – review & editing, Supervision, Investigation. **Thomas Dieterle:** Writing – review & editing, Project administration, Methodology. **Marijke Brink:** Writing – review & editing, Supervision, Methodology, Conceptualization. **Arno Schmidt-Trucksäss:** Writing – review & editing, Supervision, Methodology, Funding acquisition, Conceptualization.

## Funding

This study was supported by the Swiss National Science Foundation (185217) [to AST], the Fonds zur Förderung von Lehre und Forschung - Basel - FAG [to JMK], the SwissLife Foundation [to JMK] and the University of Basel.

## Declaration of competing interest

The authors declare the following financial interests/personal relationships which may be considered as potential competing interests: Arno Schmidt-Trucksaess reports financial support was provided by Swiss National Science Foundation. Julia Maria Kroepfl reports financial support was provided by Voluntary Academic Society (FAG Basel). Julia Maria Kroepfl reports financial support was provided by SwissLife Foundation. If there are other authors, they declare that they have no known competing financial interests or personal relationships that could have appeared to influence the work reported in this paper.
