# Supplementary material for: Heart failure with preserved ejection fraction: The role of intravascular volumes and body composition in exercise-induced progenitor cell mobilization
Source: Int J Cardiol Heart Vasc. 2026 Jun 29;65:101967. doi: 10.1016/j.ijcha.2026.101967 (PMC13330622; doi:10.1016/j.ijcha.2026.101967)
Supplement: Supplementary file 1 — Supplementary material [file mmc1.docx]

**Table 1:** Baseline characteristics of study population separated by training modality

| ***Demographic & clinical variables*** | | | |  |  | **HIT (n=15)** | | | | | **MCT (n=14)** | | |  |  |
| --- | --- | --- | --- | --- | --- | --- | --- | --- | --- | --- | --- | --- | --- | --- | --- |
| Age (at inclusion) *(mean (SD) [n])* | | | |  |  | 72.9 | | (6.2) [15] | | | 73.0 | | (9.8) [14] |  |  |
| Body mass index *(mean (SD) [n])* | | | |  |  | | 26.3 | (7.3) [15] | | | 25.8 | | (3.5) [14] |  |  |
| Systolic BP (mmHg) *(mean (SD) [n])* | | | |  |  | 135 | | (16) [15] | | | 136 | | (24) [14] |  |  |
| Diastolic BP (mmHg) *(mean (SD) [n])* | | | |  |  | 84 | | (8) [15] | | | 85 | | (14) [14] |  |  |
| NT-proBNP *(median (IQR) [n])* | | | |  |  | 265 | | (174, 497) [14] | | | 251 | | (160, 462) [14] |  |  |
| NYHA-class *(No. (%))* | | 1 | |  |  | 3 | | (20) | | | 2 | | (22) |  |  |
|  | | 2 | |  |  | 8 | | (53) | | | 6 | | (44) |  |  |
|  | | 3 | |  |  | 4 | | (27) | | | 6 | | (33) |  |  |
|  | |  | |  |  |  | |  |  | | | |  |  |  |
| ***Circulating progenitor cells*** | | | |  |  |  | |  |  | | | |  | *p-value* |  |
| CPC (cells/µl) *(mean (SD) [n])* | | | |  |  | 0.73 | | (0.63) [14] | | | 0.97 | | (0.41) [10] | *0.299* |  |
| CPC_relSSM_ (cells/µl) *(mean (SD) [n])* | | | |  |  | 0.028 | | (0.023) [14] | | | 0.034 | | (0.013) [10] | *0.513* |  |
| Total CPC count *(mean (SD) [n])* | | | |  |  | 4165193 | | (3975606) [14] | | | | 6418435 | (4603559) [9] | *0.226* |  |
| Total CPC_relSSM_ count *(mean (SD) [n])* | | | |  |  | 157660 | | (139984) [14] | | | 220348 | | (143872) [9] | *0.317* |  |
|  | | | |  |  |  | |  |  | | | |  |  |  |
| ***Intravascular volumes & hemoglobin-mass*** | | | |  |  |  | |  |  | | | |  |  |  |
| BV_relSSM_ (ml) *(mean (SD) [n])* | |  | |  |  | 202.9 | | (31.2) [15] | | | 197.3 | | (51.9) [13] | *0.725* |  |
| PV_relSSM_ (ml) *(mean (SD) [n])* | | | |  |  | 121.2 | | (19.3) [15] | | 113.6 | | | (32.2) [13] | *0.468* |  |
| RBCV_relSSM_ (ml) *(mean (SD) [n])* | | |  |  |  | 81.7 | | (13.7) [15] | | 83.6 | | | (21.4) [13] | *0.779* |  |
| Hb-mass_relSSM_ (g) *(mean (SD) [n])* | | | |  |  | 25.9 | | (3.7) [15] | | 27.3 | | | (6.8) [13] | *0.503* |  |
|  | |  | |  |  |  | |  |  | | | |  |  |  |
| ***Hematopoiesis regulation*** | |  | |  |  |  | |  |  | | | |  |  |  |
| EPO_relSSM_ (mIU/ml) *(mean (SD) [n])* | | |  |  |  | 0.39 | | (0.16) [14] | 0.47 | | | | (0.19) [14] | *0.220* |  |
| Data are presented as mean (standard deviation) or as median (IQR)*. BP* blood pressure, *NT-proBNP* B-type natriuretic peptide, *NYHA* New York Heart Association classification, *CPC* circulating progenitor cell, *BV* blood volume, *PV* plasma volume, *RBCV* red blood cell volume, *Hb* hemoglobin, *EPO* erythropoietin; Statistical comparisons between modalities were conducted using independent t-tests. | | | | | | | | | | | | | | | |
|  |  | | | | | | | | | | | | | | |
